# Utilization of evidence-based treatment in elderly patients with chronic heart failure: using Korean Health Insurance claims database

**DOI:** 10.1186/1471-2261-12-60

**Published:** 2012-07-31

**Authors:** Ju-Young Kim, Hwa-Jung Kim, Sun-Young Jung, Kwang-Il Kim, Hong Ji Song, Joong-Yub Lee, Jong-Mi Seong, Byung-Joo Park

**Affiliations:** 1 Department of Family Medicine, Seoul National University Bundang Hospital, Seoul, Korea; 2 Department of Clinical Epidemiology and Biostatics, Asan Medical Center, University of Ulsan College of Medicine, Seoul, Korea; 3 Korea Institute of Drug Safety and Risk Management, Seoul, Korea; 4 Department of Internal Medicine, Seoul National University College of Medicine, Seoul National University Bundang Hospital, Seoul, Korea; 5 Department of Family Medicine and Health Promotion Center, Hallym University Sacred Heart Hospital, Hallym University College of Medicine, Anyang-si, Korea; 6 Medical Research Collaborating Center, Seoul National University College of Medicine, Seoul National University Hospital, Seoul, Korea; 7 Department of Preventive Medicine, Seoul National University College of Medicine, Seoul, Korea

**Keywords:** Congestive heart failure, Drug utilization evaluation, Elderly, Type 2 angiotensin receptor antagonists, Angiotensin-converting enzyme antagonists, Beta-adrenergic blockers

## Abstract

**Background:**

Chronic heart failure accounts for a great deal of the morbidity and mortality in the aging population. Evidence-based treatments include angiotensin-2 receptor blockers (ARBs), angiotensin-converting enzyme inhibitors (ACE-I), beta-blockers, and aldosterone antagonists. Underutilization of these treatments in heart failure patients were frequently reported, which could lead to increase morbidity and mortality. The aim of this study was to evaluate the utilization of evidence-based treatments and their related factors for elderly patients with chronic heart failure.

**Methods:**

This is retrospective observational study using the Korean National Health Insurance claims database. We identified prescription of evidence based treatment to elderly patients who had been hospitalized for chronic heart failure between January 1, 2005, and June 30, 2006.

**Results:**

Among the 28,922 elderly patients with chronic heart failure, beta-blockers were prescribed to 31.5%, and ACE-I or ARBs were prescribed to 54.7% of the total population. Multivariable logistic regression analyses revealed that the prescription from outpatient clinic (prevalent ratio, 4.02, 95% CI 3.31–4.72), specialty of the healthcare providers (prevalent ratio, 1.26, 95% CI, 1.12–1.54), residence in urban (prevalent ratio, 1.37, 95% CI, 1.23–1.52) and admission to tertiary hospital (prevalent ratio, 2.07, 95% CI, 1.85–2.31) were important factors associated with treatment underutilization. Patients not given evidence-based treatment were more likely to experience dementia, reside in rural areas, and have less-specialized healthcare providers and were less likely to have coexisting cardiovascular diseases or concomitant medications than patients in the evidence-based treatment group.

**Conclusions:**

Healthcare system factors, such as hospital type, healthcare provider factors, such as specialty, and patient factors, such as comorbid cardiovascular disease, systemic disease with concomitant medications, together influence the underutilization of evidence-based pharmacologic treatment for patients with heart failure.

## Background

Chronic heart failure (CHF) is a significant health burden worldwide and affects approximately 10% of individuals over 65 years of age [1]. The annual incidence of new heart failure events per 1,000 individuals is 15.2 for those aged 65 to 74, 31.7 for those aged 75 to 84, and 65.2 for those over 85 years of age [1]. Korea has an aging population, and the increased incidence of age-related chronic diseases, including hypertension, diabetes, angina or other forms of cardiovascular disease, has negatively impacted the health and lives of elderly individuals.

Heart failure accounts for a great deal of the morbidity and mortality in the population, and the estimated 5-year age-adjusted mortality rate after a diagnosis of heart failure is 59% for men and 45% for women [2].

Evidence-based treatments that have been shown to decrease mortality rates [3,4] include angiotensin-2 receptor blockers (ARBs), angiotensin-converting enzyme inhibitors (ACE-I), beta-blockers, and aldosterone antagonists. However, several studies have shown that these treatments are underutilized and are often prescribed at lower dose levels [5-10]. Factors related to the underutilization of evidence-based treatments include a lack of initiation or early discontinuation. The causes of this lack of initiation include the contraindication of drug use, lack of knowledge, lack of expertise for using these drugs, lack of time, and economic restraints [9]. However, in many cases, the reasons for underutilizing evidence-based therapy are unclear. Some evidence has been collected from several randomized clinical trials (RCTs), but most RCTs have limited generalizability for treatment options because these trials typically have strict inclusion and exclusion criteria. Euro Heart surveys found that among 10,701 heart failure patients, only 1,346 were eligible to participate in the majority of RCTs [11]. However, these findings do not explain the underutilization of evidence-based treatment in eligible individuals, and elderly CHF patients could significantly benefit from evidence-based treatment [12,13].

The causes of treatment underutilization may include the fear of polypharmacy, inaccurate perceptions concerning adverse effects, and contraindications. However, the mortality rate of patients with renal insufficiency was lower for those who received ACE-I, beta-blockers, statins, and aspirin [14]. Beta-blockers can be prescribed safely to patients with diabetes [15-17] and chronic obstructive pulmonary disease [18-21], and this treatment significantly decreases the morbidity and mortality caused by heart failure. Also, the utilization of evidence-based treatment for patients with heart failure can significantly reduce heart failure-related hospital admission and mortality [12,22,23].

We evaluated the utilization of evidence-based treatments in elderly patients with heart failure using a claims database so as to assure generalizability for elderly individuals. We also studied the patient and provider factors that were related to the utilization of each drug.

## Methods

### Data source

Patients were identified from the Korean Health Insurance Review and Assessment Service database (KHIRA), which contains medical claims data for the entire Korean population [24] as a result of the National Insurance Health System. Patients pay an average of 30% of the total medical costs related to almost all diseases. Healthcare providers submit reports concerning the medical services performed to the KHIRA for a review of the medical costs incurred. These reports contain information on the diagnosis that has been coded in accordance with the International Classification of Diseases Tenth Revision [ICD-10] as well as information related to outpatient or inpatient status, drug name, dosage, prescription date, duration, and method of administration. The KHIRA provided data with the individual identifier removed, in accordance with the Act on the Protection of Personal Information Maintained by Public Agencies. Thus, the database included an unidentifiable code representing each individual with data concerning the patients’ age, gender, diagnosis and lists of prescribed drugs.

The database contained information regarding 1,093,262 elderly patients aged above 65 years and 11,842,586 prescriptions from January 1, 2005, to June 30, 2006.

This study was approved by the institutional review board of the Seoul National University Bundang Hospital with reference number of B-1011-115-105.

### Study population

We identified older adults who were over the age of 65 and had been hospitalized with a primary discharge diagnosis of heart failure (ICD-10 codes: I11, I13, and I50) between January 1, 2005, and June 30, 2006. Though diagnosis of heart failure by ICD-10 codes in the KHIRA database was not validated, we tried to increase diagnostic accuracy by adjusting medications for heart failure such as digoxin, inotropics and diuretics.

We excluded patients if they had a length of stay less than 24 hours or did not have medication records. In total, 28,922 patients were admitted with a primary discharge diagnosis of heart failure.

### Prescription of evidence-based treatment in CHF

Three classes of prescription medications were evaluated based on evidence-based treatment: ACE-I or ARB (group A), beta-blockers (group B), and aldosterone antagonists (Aldo group).

The utilization of evidence-based treatments was defined as treatments that were prescribed after hospitalization for heart failure. We prioritized groups A and B rather than the Aldo group because the 2005 American College of Cardiology and American Heart Association (ACC/AHA) heart failure guidelines with the 2009 focused update [25] recommended the addition of an aldosterone antagonist for the treatment of patients with moderate to severe HF and the reduced ejection fraction who could be carefully monitored for preserved renal function and normal plasma potassium concentrations. Therefore, we classified the evidence-based treatment groups as A, B, Aldo, and A + B. The A + B group was assigned if a patient received both A and B group treatments. If a patient received A, B, and Aldo group treatments, then the patient was assigned to the A + B group. If a patient received A and Aldo group treatments, then the patient was assigned to group A. If a patient only received Aldo group treatment, then the patient was assigned to the Aldo group. Group B consisted of patients who were given only a beta-blocker treatment or beta-blockers in addition to aldosterone antagonists.

### Covariates

Data concerning patient age, gender, area of residence, type of prescription resources from inpatient or outpatient clinic and the utilization of hospital type (primary care clinics, secondary hospitals, which typically refer to large, community but non-teaching hospitals, or tertiary hospitals, which usually refer to a teaching or university hospital) were obtained from the database. Previous cardiovascular disease histories were collected using the diagnosis codes for angina pectoris (I20), myocardial infarction (I21-22 and I25.2), transient ischemic attack or ischemic stroke (G45-46, H34, and I60-69), peripheral artery disease (I70-71, I73.1, I73.8-73.9, I77.1, I79.0, I79.2, K55.1, K55.8-55.9, and Z95.8-95.9), atrial fibrillation or flutter (I48), and valvular heart disease (I01, I05-09, and I34-39).

We also included any medical histories of hypertension (I11-I13 an d I15), hyperlipidemia or dyslipidemia (E78), end-stage renal disease (I12.0, I13.1, N03.2-03.7, N05.2, N05.7, N18-19, N25.0, Z49.0-49.2, Z94.0, and Z99.2), chronic lung disease (I27.8, I27.9, J40-47, J68.4, J70.1, and J70.3), chronic liver disease (B18, K70, K71, K73-74, K76, and Z94.4), systemic cancer (C00-C26, C30-C34, C37-C41, C43, C45-58, C60-85, C88, and C90-97), dementia (F00-F03, F05.1, G30, and G31.1) and depression (F32-33).

Concomitant medication use was adjusted in the model. Concomitant drugs included heart failure medications, such as diuretics, calcium channel blockers, nitrates, digoxin, amiodarone, and hydralazine, and lipid-lowering medications, such as statins, fibrates, and ezetimibe. Anti-diabetic medications were also included, such as sulfonylurea, metformin, alpha glucosidase inhibitors, thiazolidinediones, and insulin.

### Statistical analysis

Statistical analyses were performed using SAS software, version 9.1 (Cary, North Carolina).

We evaluated baseline characteristics with previous cardiovascular disease, medications and other systemic medical histories between each group of evidence based treatment and non use group using Student’s *t* test for continuous variable and chi-square test for categorical variables,

Multivariable logistical regression model was used to evaluate clinical factors associated with each evidence-based group. The model incorporated the following demographic factors (age, gender, residence area, utilization of hospital type, specialty of health care providers and type of prescription resources), previous cardiovascular diseases (angina, myocardial infarction, valvular heart disease, atrial fibrillation or flutter, transient ischemic attack), systemic medical diseases (hypertension, hyperlipidemia, chronic lung disease, end stage renal disease) and concomitant medications (heart failure medication, antidiabetic drugs) by forward selection methods. We also performed the similar multivariable logistic regression analysis in subgroup who were treated with both digoxin and diuretics, which could indicate patients with symptom relieving treatment for heart failure. Subgroup analysis was shown for the purpose of increasing diagnostic accuracy for heart failure.

## Results

### Study population

**Figure 1:**
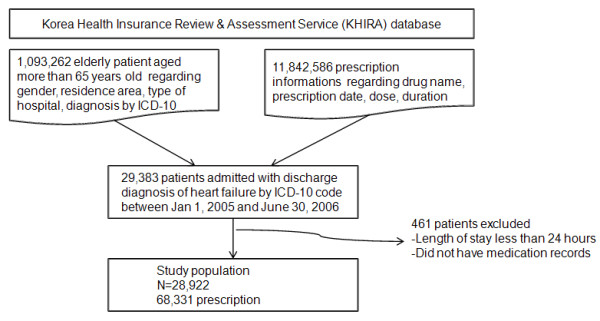
Selection of study population. ICD-10: International Classification of Disease, Tenth Revision.

A total of 29,104 patients were admitted with a primary diagnosis of congestive heart failure during the study period, although 182 patients had no medical information recorded. Therefore, 28,922 patients were analyzed for this study concerning the utilization of evidence-based treatments for congestive heart failure and flow of study population was represented in Figure1. The baseline characteristics of the study population are shown in Table1.

**Table 1:** Clinical characteristics related to the utilization of disease-modifying treatments in the study population

	**Total study population**	**ACEI or ARB and Beta-blockers**	**ACEI or ARB**	**Beta-blockers**	**Aldosterone antagonist**	**None**
	**(N = 28922)**	**(N = 6261)**	**(N = 9540)**	**(N = 2837)**	**(N = 2007)**	**(N = 8277)**
	**N (%)**	**21.7% total**	**33.0% total**	**9.8% total**	**6.9% total**	**28.6% total**
Mean age (SD)	77.5 (7.0)	76.7 (6.8)*	77.7 (7.0)	76.8 (6.7)*	78.4 (6.9)	77.9 (7.2)
Age group, y						
65-74	10296 (35.6)	2477 (39.6)*	3299 (34.6)	1117 (39.4)**	604 (30.1)*	2799 (33.8)
75-84	13776 (47.6)	2929 (46.8)	4563 (47.8)	1341 (47.3)	1024 (51.0)	3919 (47.4)
85-	4850 (16.8)	855 (13.7)	1678 (17.6)	379 (13.4)	379 (18.9)	1559 (18.8)
Sex						
Women	20927 (72.4)	4420 (70.6)*	6885 (72.2)	2123 (74.8)*	1489 (74.2)	6010 (72.6)
Healthcare provider specialty						
Internal medicine	27035 (93.5)	6028 (96.3)**	9108 (95.5)**	2651 (93.4)**	1853 (92.3)**	7395 (89.3)
Others	1887 (6.5)	233 (3.7)	432 (4.5)	186 (6.6)	154 (7.7)	882 (10.7)
Type of hospital						
Primary hospital	372 (3.0)	55 (0.9)**	188 (2.0)**	102 (3.6)**	86 (4.3)**	441 (5.3)
Secondary hospital	9801 (33.9)	1035 (16.5)	2800 (29.6)	1035 (36.5)	1018 (50.7)	3913 (47.3)
Tertiary hospital	18249 (63.1)	5171 (82.6)	6552 (68.7)	1700 (59.9)	903 (45.0)	3923 (47.4)
Residence area						
Urban	15441 (53.4)	3994 (63.8)**	5384 (56.4)**	1435 (50.6)*	778 (38.8)**	3850 (46.5)
Rural	13481 (46.6)	2267 (36.2)	4156 (43.6)	1402 (49.4)	1229 (61.2)	4427 (53.5)
Source of prescription						
Outpatient	22046 (76.2)	5165 (82.5)	8295 (86.9)	2385 (84.1)	1731 (86.2)	4470 (54 )
Cardiovascular disease						
Angina	4413 (15.3)	1378 (22.0)**	1485 (15.6)**	509 (17.9)**	193 (17.9)	848 (10.3)
Myocardial infarction	3078 (10.6)	981 (15.7)**	1049 (11.0)**	289 (10.2)**	141 (7.0)	618 (7.5)
Transient ischemic stroke	4609 (15.9)	1027 (16.4)	1364 (14.3)**	515 (18.2)	325 (16.2)	1378 (16.7)
Peripheral artery disease	1255 (4.3)	329 (5.3)*	379 (4.0)	141 (5.0)*	70 (3.5)	336 (4.1)
Arterial fibrillation or flutter	5720 (19.8)	1780 (28.4)**	2089 (21.9)**	567 (20.0)**	362 (18.0)**	922 (11.1)
Valvular heart disease	2548 (8.8)	846 (13.5)**	981 (10.3)**	197 (6.9)**	137 (6.8)**	387 (4.7)
Medical history						
Hypertension	11394 (39.4)	2149 (34.3)**	3756 (39.4)*	1364 (48.1)**	670 (33.4)**	3455 (41.7)
Diabetes mellitus	9882 (34.2)	2607 (41.6)**	3454 (36.2)**	892 (31.4)*	591 (29.5)	2338 (28.3)
Hyperlipidemia or dyslipidemia	6624 (22.9)	2041 (32.6)**	2279 (23.9)**	637 (22.5)**	349 (17.4)	1318 (15.9)
End-stage renal disease	1884 (6.5)	622 (9.9)**	574 (6.0)*	183 (6.5)*	97 (4.8)	408 (4.9)
Chronic lung disease	9829 (34.0)	2041 (32.6)	3516 (36.9)**	850 (30.0)	832 (41.5)**	2610 (31.5)
Chronic liver disease	4474 (15.5)	983 (15.7)	1521 (15.9)	423 (14.9)	325 (16.2)	1222 (14.8)
Systemic cancer	1389 (4.8)	329 (5.3)*	493 (5.2)	120 (4.2)	84 (4.2)	363 (4.4)
Dementia	2000 (6.9)	320 (5.1)**	548 (5.7)**	210 (7.4)*	161 (8.0)	761 (9.2)
Depression	1517 (5.3)	334 (5.3)	477 (5.0)	153 (5.4)	108 (5.4)	445 (5.4)
Concomitant medication						
Diuretics	20810 (76.7)	5484 (88.2)**	7886 (84.1)**	1958 (71.0)**	1702 (86.8)**	3780 (55.6)
Calciumchannel blockers	9944 (36.7)	2408 (38.7)	3139 (33.5)*	1224 (44.4)**	542 (27.7)**	2631 (38.7)
Nitrates	7282 (26.9)	2546 (41.0)**	2514 (26.8)**	795 (28.8)**	352 (18.0)*	1075 (15.8)
Digoxin	11913 (43.9)	2918 (46.9)**	4569 (48.7)**	1072 (38.9)**	1036 (52.9)**	2318 (34.1)
Inotropics	5286 (19.5)	1660 (26.7)**	1813 (19.3)*	358 (13.0)**	282 (14.4)*	1173 (17.2)
Amiodarone	986 (3.6)	359 (5.8)**	353 (3.8)**	94 (3.4)*	37 (1.9)	143 (2.1)
Hydralazine	427 (1.6)	141 (2.3)**	116 (1.2)	55 (2.0)*	22 (1.1)	93 (1.4)
Lipid lowering agents	4465 (16.5)	1667 (26.8)**	1509 (16.1)**	459 (16.6)**	175 (8.9)	655 (9.6)
Anti-diabetic medication	6453 (23.8)	1851 (29.8)**	2302 (24.5)**	600 (21.8)*	374 (19.1)	1326 (19.5)
Anti-thrombotics	17473 (64.4)	5024 (80.8)**	6299 (67.2)**	1860 (67.4)**	1050 (53.6)**	3240 (47.6)

Data for categorical variables are expressed as n (%) and quantitative variables as means (standard deviation). SD: standard deviation.

Variables in each group of evidence based treatments were compared with non user group as control group using chi-square methods or Student’s t test methods.

*p < 0.05, statistically differences, **p < 0.01, statistically differences.

The mean age at the time of admission was 77.5 ± 7.0 years; 64.4% of patients were more than 75 years of age, and 72.4% of patients were female. Most patients were admitted to tertiary hospitals, and the coexisting cardiovascular diseases included atrial fibrillation or flutter (19.8%), transient ischemic attack (15.9%), and angina (15.3%). Common comorbidities included hypertension (39.4%), diabetes (34.2%), and chronic obstructive lung disease (34.0%).

### Utilization of evidence-based treatment in elderly CHF patients

In total, 71.4% of elderly heart failure patients received evidence-based treatment. For each treatment group analysis, the A + B group comprised 21.7% of the total patient group, group A made up 33.0%, group B 9.8%, and the Aldo group represented 6.9% of the total study population.

Females composed 70% of all study patients, and the mean age of each group was between 76 and 79 years of age.

For the A + B group, the specialty of 96% of the healthcare providers was internal medicine, and 82.6% of the A + B patients were treated at tertiary hospitals. However, the specialty of 83% of healthcare providers for patients who were not given evidence-based treatment (non-use group) was internal medicine, and 47.4% of these patients were treated at tertiary hospitals.

Patients in the A + B group had higher rates of angina, myocardial infarction, atrial fibrillation, valvular heart disease, and diabetes compared to those in the non-use group. However, dementia was more pronounced in the non-use group. Patients in the A + B group were more likely to receive symptom-relieving heart failure drugs, such as diuretics, digoxin, and other inotropics, than were patients in the non-use group.

### Factors associated with the utilization of each evidence-based treatment

Factors associated with the utilization of evidence-based treatment were dependent on admission to a tertiary hospital, having a more specialized healthcare provider, prescribed from outpatient clinic, atrial fibrillation, valvular heart disease and the administration of more symptom-relieving treatments, such as diuretics and digoxin.

For the A + B group as shown in Table2, associated factors for utilizing evidence-based treatment included admission to a tertiary hospital, healthcare provider specialty, prescription from outpatient clinic, the diagnosis of angina, myocardial infarction, atrial fibrillation or valvular heart disease, and the administration of inotropics, antithrombotic agents, nitrate, or lipid-lowering agents. Chronic lung disease was negative associated factor in utilization of A + B group and B group, while it was positive associated factor in A group and Aldo group.

**Table 2:** Multivariable logistic regression for factors associated with the utilization of disease-modifying treatments in the entire study population of 28, 922 patients with chronic heart failure

**Predictors of utilization**	**Disease-modifying treatment group**	**ACEI or ARB and Beta-blockers**	**ACEI or ARB**	**Beta-blockers**	**Aldosterone antagonist**
	**(71.4% total)**	**(21.7% total)**	**(33.0% total)**	**(9.8% total)**	**(6.9% total)**
	**aPR (95% CI)**	**aPR (95% CI)**	**aPR (95% CI)**	**aPR (95% CI)**	**aPR (95% CI)**
Older Age group		0.92 (0.89-0.97)	1.09 (1.05-1.14)	0.86 (0.81-0.91)	1.08 (1.01-1.16)
Women				1.13 (1.03-.1.25)	
Specialty of healthcare providers being internal medicine	1.26 (1.12--1.54)	1.20 (1.13-1.39)	1.28 (1.08-1.42)		0.82 (0.68-0.98)
Tertiary hospital	2.07 (1.85-2.31)	1.95 (1.81-2.11)	1.15 (1.09-1.22)	0.86 (0.79-0.93)	0.56 (0.52-0.62)
Urban residence	1.37 (1.23-1.52)	1.08 (1.00-1.15)	1.19 (1.12-1.26)		0.67 (0.61-0.75)
Outpatient prescription	4.02 (3.31-4.72)	2.76 (2.56-2.97)	5.02 (4.64-5.47)	3.08 (2.65-3.56)	3.39 (2.83-4.03)
Cardiovascular disease					
Angina	1.40 (1.38-1.47)	1.12 (1.02-1.20)	0.90 (0.83-0.97)	1.21 (1.08-1.35)	0.78 (0.67-0.92)
Myocardial infarction	1.37 (1.28-1.45)	1.11 (1.04-1.23)			0.81 (0.67-0.97)
Transient ischemic stroke	0.82 (0.71-0.94)		0.85 (0.78-0.91)		1.24 (1.09-1.41)
Peripheral artery disease			0.86 (0.75-0.98)		
Arterial fibrillation or flutter	1.36 (1.20-1.56)	1.26 (1.17-1.36)	0.87 (0.81-0.98)	1.17 (1.05-1.31)	
Valvular heart disease	1.28 (1.10-1.53)	1.26 (1.14-1.39)		0.76 (0.65-0.89)	0.78 (0.65-0.95)
Medical history					
Hypertension			1.09 (1.03-1.15)	1.20 (1.11-1.31)	0.63 (0.57-0.70)
Diabetes mellitus	1.26 (1.14-1.40)	1.08 (1.02-1.16)	1.10 (1.04-1.16)	0.80 (0.74-0.88)	
Hyperlipidemia or dyslipidemia				0.85 (0.77-0.94)	
End-stage renal disease		1.30 (1.16-1.45)	0.78 (0.70-0.88)		
Chronic lung disease	0.67 (0.54-0.87)	0.83 (0.77-0.89)	1.13 (1.07-1.19)		1.31 (1.19-1.45)
Dementia			0.83 (0.75-0.92)	0.81 (0.74-0.88)	
Concomitant medication					
Diuretics	3.41 (3.21-3.65)	1.87 (1.71-2.05)	1.29 (1.13-1.31)	0.82 (0.77 -0.92)	2.24 (1.94-2.58)
CCB		1.18 (1.11-1.26)	0.87 (0.82-0.93)		0.70 (0.63-0.78)
Nitrates	1.79 (1.61-2.01)	1.51 (1.41-1.62)	0.85 (0.79-0.91)	1.30 (1.19-1.42)	0.75 (0.66-0.86)
Digoxin	1.32 (1.24-1.53)		1.23 (1.17-1.30)		1.28 (1.19-1.37)
Hydralazine	1.60 (1.09-2.38)	1.61 (1.28-2.02)	0.73 (0.58-0.92)	1.12 (1.02-1.28)	
Inotropics	1.59 (1.41-1.79)	1.43 (1.33-1.54)	0.92 (0.86-0.98)		
Lipid-lowering agents	1.80 (1.57-2.07)	1.64 (1.52-1.77)	0.84 (0.78-0.90)	0.84 (0.78-0.92)	0.65 (0.55-0.77)
Anti-diabetic medication	1.92 (1.80-2.05)	1.12 (1.04-1.20)			0.65 (0.58-0.72)
Anti-thrombotics	1.88 (1.69-2.09)	1.78 (1.65-1.92)		0.70(0.62-0.79)	

Adjusted for demographic factors (age, gender, residence area, utilization of hospital type, specialty of health care providers and type of prescription resources), previous cardiovascular diseases (angina, myocardial infarction, valvular heart disease, atrial fibrillation or flutter, transient ischemic attack), systemic medical diseases (, hypertension, hyperlipidemia, chronic lung disease, end stage renal disease) and concomitant medications (heart failure medication, antidiabetic drugs).

aPR; adjusted Prevalent Ratio, CI: Confidence Interval, CCB: Calcium channel blockers.

For group A, associated factors for utilization were similar to those of the A + B group and included admission to a tertiary hospital, healthcare provider specialty and prescription from outpatient clinic, but patients with end-stage renal disease were less likely to receive ACEi or ARB treatment.

For group B, relatively young patients and those admitted to a secondary hospital were more likely to receive beta-blockers, and patients with diabetes or chronic lung disease were less likely to receive treatment. Digoxin, diuretics, or inotropic agent use were negative associated factors in group B.

In the Aldo group, patients who were admitted to primary or secondary hospital, those with dementia or transient ischemic attack, and those living in rural areas were more likely to be treated with only aldosterone antagonists.

Data from the subgroup of our study population that was given digoxin and diuretics as compared to those data from the evidence-based treatment group and the non-use group are shown in Table3. Results from the subgroup analysis were similar as in total study population. For A + B subgroup, admission to tertiary hospital, prescription from outpatient clinic, having myocardial infarction, atrial fibrillation or valvular heart disease and prescribed with inotropics, antidiabetic medication or anti thrombotic medication were positive associated factors. Chronic lung disease was negative associated factor for utilization of beta blocker treatment in our subgroup analysis. End stage renal disease was negative associated factor in A subgroup. Beta blocker treatment subgroup were relatively young and admission to tertiary hospital was negative associated factor.

**Table 3:** Multivariable logistic regression for factors associated with the utilization of disease-modifying treatment in 10,091 individuals administered digoxin and diuretics for chronic heart failure

**Predictors of utilization**	**Disease-modifying treatments group**	**ACEI or ARB and Beta-blockers**	**ACEI or ARB**	**Beta-blockers**	**Aldosterone antagonist**
	**(84.8% total)**	**(26.9% total)**	**(40.4% total)**	**(8.5% total)**	**(9.0% total)**
	**aPR (95% CI)**	**aPR (95% CI)**	**aPR (95% CI)**	**aPR (95% CI)**	**aPR (95% CI)**
Older Age group		0.91 (0.84-0.97)	1.07 (1.01-1.14)	0.87 (0.78-0.96)	
Women		1.16 (1.05-1.29)			0.83 (0.71-0.97)
Specialty of healthcare providers being internal medicine	1.37 (1.09-1.71)		1.23 (1.01-1.49)		
Tertiary hospital	1.78 (1.49-2.12)	1.94 (1.73-2.16)	1.12 (1.03-1.22)	0.78 (0.68-0.88)	0.55 (0.48-0.64)
Urban residence	1.46 (1.23-1.74)		1.19 (1.09-1.29)		0.72 (0.62-0.84)
Outpatient prescription	3.87(2.95-4.57)	2.19 (1.95-2.46)	3.17 (2.75-3.64)	2.18 (1.80-2.64)	2.30 (1.69-3.05)
Cardiovascular disease					
Myocardial infarction		1.19 (1.03-1.36)			
Transient ischemic stroke			0.78 (0.68-0.86)		1.51 (1.25-1.83)
Arterial fibrillation or flutter	1.54 (1.28-1.84)	1.37 (1.24-1.51)	0.90 (0.83-0.99)		
Valvular heart disease		1.23 (1.08-1.41)			0.71 (0.54-0.93)
Medical history					
Hypertension			1.10(1.02-1.22)		0.62 (0.53-0.73)
Diabetes mellitus	1.32 (1.10-1.56)		1.09 (1.01-1.20)		0.84 (0.71-0.98)
Hyperlipidemia or dyslipidemia				0.78 (0.65-0.93)	
End-stage renal disease			0.78 (0.68-0.86)		
Chronic lung disease		0.84 (0.76-0.93)	1.13 (1.03-1.23)	0.86 (0.74-0.93)	1.15 (1.00-1.34)
Dementia	0.61 (0.42-0.90)		0.84 (0.70-0.99)		1.41 (1.09-1.82)
Depression					
Concomitant medication					
CCB*		1.28 (1.15-1.42)	0.82 (0.75-0.90)	1.31 (1.13-1.53)	0.76 (0.64-0.90)
Nitrates	1.39 (1.21-1.60)	1.33 (1.20-1.47)			0.73 (0.61-0.87)
Inotropics	1.78 (1.48-2.16)	1.53 (1.37-1.69)		0.72 (0.60-0.86)	0.68 (0.56-0.83)
Hydralazine		1.64 (1.14-2.37)			
Lipid-lowering agents	1.93 (1.52-2.48)	1.61 (1.43-1.83)			0.46 (0.34-0.62)
Anti-diabetic medication		1.14 (1.01-1.25)			
Anti-thrombotics	1.63 (1.35-1.96)	1.55 (1.37-1.75)			0.59 (0.49-0.67)

Adjusted for demographic factors (age, gender, residence area, utilization of hospital type, specialty of health care providers and type of prescription resources), previous cardiovascular diseases (angina, myocardial infarction, valvular heart disease, atrial fibrillation or flutter, transient ischemic attack), systemic medical diseases (hypertension, hyperlipidemia, chronic lung disease, end stage renal disease) and concomitant medications (heart failure medication, antidiabetic drugs).

aPR; adjusted Prevalent Ratio, CI: Confidence Interval, CCB: Calcium channel blockers.

## Discussion

This nationwide study revealed the utilization patterns of evidence-based pharmacologic treatment among elderly heart failure patients. Our study indicated that evidence-based treatment is underutilized in elderly heart failure patients, which is similar to the findings of many other studies.

The evidence-based treatment group was more likely to be admitted to tertiary hospitals, and their healthcare providers were more specialized than those of patients in the non-use group. Although we did not find the subspecialty of internal medicine in our data set, we hypothesized that most internal medicine care providers would be cardiologists. The evidence-based treatment group was more likely to have coexisting cardiovascular diseases, such as angina, myocardial infarction, atrial fibrillation, and valvular heart disease, and comorbidities, such as diabetes and hyperlipidemia.

The non-use group was more likely to have been admitted to a primary or secondary hospital and treated by less specialized healthcare providers and was also more likely to have dementia and living in rural area. And rate of prescriptions from outpatient setting in non use group was 54%, which was lower than that of evidence based treatment group, where rate was 76%. Therefore, it could be inferred that the non-use group was more likely to be institutionalized. Shibata et al [10]., studied the utilization pattern of evidence-based treatment for heart failure in institutionalized elderly patients and found prescription rates to be low; the frequency of ACEi and ARB treatment was 51%, and the frequency of beta-blocker treatment was 16%.

When considering the associated factors for evidence-based treatment in elderly heart failure patients, the specialty of the treating healthcare providers and the type of hospital were important factors. This may be related to medication costs and reimbursements. If a patient is institutionalized due to dementia, the percentage of the fee that the national healthcare insurance system covers is limited, and the hospital should thus attempt to reduce medication and examination costs. Another factor related to the utilization of evidence-based treatment may be the healthcare providers. Beta-blockers have been traditionally misunderstood as aggravating chronic obstructive pulmonary disease or poor glycemic control in diabetes. However, several studies [26,27] have suggested that patients with chronic obstructive pulmonary disease tolerate selective beta-blockers well, although these medications should be administered at the lowest possible dose and require close monitoring. Carvedilol, the most frequently used beta-blocker for patients with heart failure, can also be safely prescribed to diabetic patients [28] because it has neutral effects on blood glucose levels. Many studies have shown that evidence-based beta-blockers, such as carvedilol or nebivolol [29], should be prescribed for diabetic heart failure patients because these treatments have significant health benefits for all causes of mortality. Our data suggest that many physicians appear reluctant to prescribe beta-blockers for patients with chronic lung disease or diabetes.

Many researchers from other countries have studied the utilization patterns of evidence-based treatment in heart failure. Research using the Medicare database [12] for elderly populations with heart failure found that the prescription rate of those prescribed only ACEi or ARB treatment was 27.9%. The rate of beta-blocker prescription was 15.7%, and patients were prescribed both treatments at a rate of 28.4%. Data from the Registry to Improve the Use of Evidence-Based Heart Failure Therapies in the Outpatient Setting (IMPROVE HF) [30] indicated that the prescription rate for ACEi or ARBs was 79.3%, and that for beta-blockers was 85.8%. This suggested that the utilization patterns of evidence-based treatment are dependent on the database that is used for the analysis. For comparability with our data, we searched articles using claims databases.

Gislason et al [31]., studied the persistent use of evidence-based pharmacotherapy in heart failure using the Danish National Patient Registry claims database. Prescription was defined as treatment initiation after discharge from a hospital admission due to heart failure, which was similar to our study design. The prescription rate for ACEi or ARB treatment was 43%. Beta-blockers were prescribed at a rate of 27%, while spironolactone was prescribed at a rate of 19%.

Another study was published using a population-based cohort (1999–2001) of 9,942 patients with heart failure who had been hospitalized in Ontario, Canada [32]. These researchers investigated the prescription rates of evidence-based treatment for patients admitted to the hospital due to heart failure after their discharge. The prescription rate of ACEi or ARB treatment was approximately 77%, while that of beta-blockers was 33%; however, the authors stratified prescription rates with respect to the validated heart failure risk scores. One study [9] used the Euro Heart Survey on Heart Failure, which included 46,788 heart failure patients from 115 hospitals in 24 ESC member countries. These data showed that the prescription rate for ACEi or ARB treatment was 62%, and the beta-blocker prescription rate was 37%.

**Figure 2:**
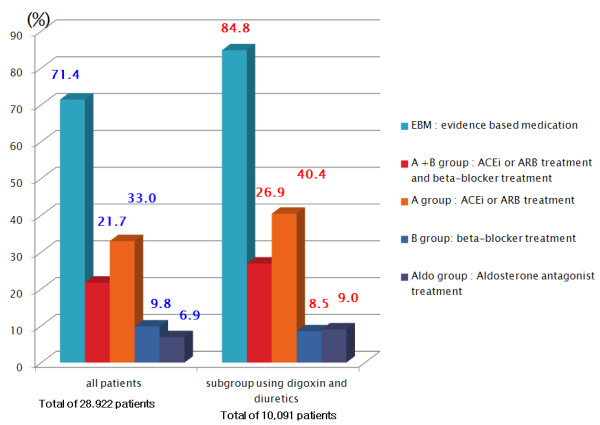
Rates of evidence-based treatment in elderly heart failure patients.

The prescription rate of ACEi or ARB treatment was 54.7% for our total study population and 67.3% when the population using diuretics and digoxin was analyzed, as shown in Figure2. Similarly, the prescription rate of beta-blockers was 31.5% for the total study population and 35.4% in the population using diuretics and digoxin. Some of the underutilization in beta-blockers could be due to recent hospitalization of heart failure patients, because stabilization of patient’s status should be prior to initiating beta blockers.

Our data suggested that the non-use group may consist of institutionalized patients with dementia and that healthcare system factors, such as medication cost and reimbursement, healthcare provider factors, such as specialty or knowledge, and patient factors, such as comorbidities, could contribute to the underutilization of evidence-based pharmacologic treatment in heart failure.

We used the KHIRA claims database to ensure high generalizability and eliminate recall and selection bias. Our study results reflected the actual heart failure population, and because we used the National Health Insurance System, we were able to obtain detailed information concerning the medications that were used to treat diseases coded by the International Classification Codes. Thus, we were able to estimate the non-use group.

However, our study had several limitations. First, the diagnosis of heart failure in the KHIRA database was not validated, although a validity study that compared diagnoses by ICD-10 codes to clinical information obtained using the medical records showed a positive predictive value of approximately 70% [33].

Second, our study was limited because data concerning the left ventricular ejection fraction were not available. Therefore, our population had potentially considerably more diverse clinical characteristics, although we attempted to adjust for this limitation by including many comorbidities and medications. We also analyzed subgroups according to digoxin and diuretic use, which represented symptom-relieving heart failure treatment, and the results did not differ.

Third, the contraindication of evidence-based treatment could not be clearly defined. In the non-use group, long-term bedridden status may contraindicate beta-blocker utilization or cause patients to be intolerable state in lowering their blood pressure.

## Conclusions

In conclusion, an underutilization of evidence-based treatments was observed in elderly Korean heart failure patients. Coexisting cardiovascular disease severity and concurrent medications were associated with a greater use of evidence-based treatment. The specialty of the healthcare providers, prescription from outpatient clinic and the hospital type were important factors for utilizing evidence-based treatment. Our results could be related to the healthcare system, various healthcare provider factors and patient factors.

Further research should focus on improving the quality of treatment programs for elderly heart failure patients.

## Competing interests

The authors declare that there is no competing interests.

## Authors’ contributions

J.Y.Kim jointly conceived the study with H.J.Kim, designed the study plan and prepared the manuscript; S.Y.Jung conducted the statistical analysis, and edited the manuscript; K.I. Kim and H.J.Song supervised its analysis and edited the manuscript; J.Y.Lee and J.M.Seong carried out the analysis of subpopulation group and edited the manuscript; B.J. Park supervised design and analysis of the study and edited the discussion part. J.Y.Kim and H.J.Kim equally contributed to this paper as first co-authors. All authors read and approved the final manuscript.

## Pre-publication history

The pre-publication history for this paper can be accessed here:

http://www.biomedcentral.com/1471-2261/12/60/prepub
